# Reordering based integrative expression profiling for microarray classification

**DOI:** 10.1186/1471-2105-13-S2-S1

**Published:** 2012-03-13

**Authors:** Xiaogang Wu, Hui Huang, Madhankumar Sonachalam, Sina Reinhard, Jeffrey Shen, Ragini Pandey, Jake Y Chen

**Affiliations:** 1 School of Informatics, Indiana University, Indianapolis, IN 46202, USA; 2 Indiana Center for Systems Biology and Personalized Medicine, Indiana University, Indianapolis, IN 46202, USA; 3 MedeoLinx, LLC, Indianapolis, IN 46280, USA

## Abstract

**Background:**

Current network-based microarray analysis uses the information of interactions among concerned genes/gene products, but still considers each gene expression individually. We propose an organized knowledge-supervised approach - Integrative eXpression Profiling (IXP), to improve microarray classification accuracy, and help discover groups of genes that have been too weak to detect individually by traditional ways. To implement IXP, ant colony optimization reordering (ACOR) algorithm is used to group functionally related genes in an ordered way.

**Results:**

Using Alzheimer's disease (AD) as an example, we demonstrate how to apply ACOR-based IXP approach into microarray classifications. Using a microarray dataset - GSE1297 with 31 samples as training set, the result for the blinded classification on another microarray dataset - GSE5281 with 151 samples, shows that our approach can improve accuracy from 74.83% to 82.78%. A recently-published 1372-probe signature for AD can only achieve 61.59% accuracy in the same condition. The ACOR-based IXP approach also has better performance than the IXP approach based on classic network ranking, graph clustering, and random-ordering methods in an overall classification performance comparison.

**Conclusions:**

The ACOR-based IXP approach can serve as a knowledge-supervised feature transformation approach to increase classification accuracy dramatically, by transforming each gene expression profile to an integrated expression files as features inputting into standard classifiers. The IXP approach integrates both gene expression information and organized knowledge - disease gene/protein network topology information, which is represented as both network node weights (local topological properties) and network node orders (global topological characteristics).

## Background

Network-based gene expression analysis has been proposed for candidate biomarker discovery by integrating disease susceptibility genes, gene expressions, and gene/protein interaction networks[1,2]. Current network-based gene expression analysis methods do utilize the information of the interactions among concerned genes or gene products, but they still consider each single gene expression individually, without taking into account the expression values of neighbor genes with similar or related functions in a given network.

We propose a concept - *Integrative eXpression Profiling *(*IXP*), which can not only improve microarray classification accuracy by serving as a feature transformation approach, but also help in the discovery of groups of genes that have been too weak to detect individually through traditional methods. Functionally related genes individually expressed with lower differentials, which have often been considered as noise and ignored in traditional studies, can be readily identified by virtue of their coordinate expression within IXP profiles. To implement IXP, we need first to group functionally related genes together in an ordered way. Traditional network analyses often fail to find patterns in ranked or clustered adjacency matrix of a network when facing complex networks having higher inseparability, where no "clear cluster" or no "absolute rank" exists. Here we use the ant colony optimization reordering (ACOR) algorithm [3,4], instead of conventional network-based gene ranking [5], or graph clustering [6]. In the ACOR algorithm, the task of reordering nodes is represented as the problem of finding optimal density distributions of "ant colonies" on all nodes of the network, in which simulated ants roam all possible network paths iteratively. According to this density distribution, the adjacency matrix of the network with ranked nodes is shown as a map in order to reveal the system-level features of the network. The ACOR algorithm has been tested in both yeast protein networks [4] and human disease protein networks [3].

In this work, we use Alzheimer's disease (AD) as a case study, to illustrate how to apply the ACOR-based IXP approach to the blinded classification on a microarray dataset - GSE5281 with 151 samples (testing set, 67 controls and 84 AD patients), by using another much smaller microarray dataset - GSE1297 with 31 samples (9 controls and 22 AD patients) as training set. The result for the blinded classification on GSE5281 shows that our approach can improve accuracy from original 74.83% to 82.78% by using SVM classifier. A recently-published 1372-probe signature for AD [7]can only achieve 61.59% accuracy in the same condition. The ACOR-based IXP approach also performs better than the IXP approach based on ranking, clustering, and random-ordering in an overall performance comparison.

## Methods

**Figure 1:**
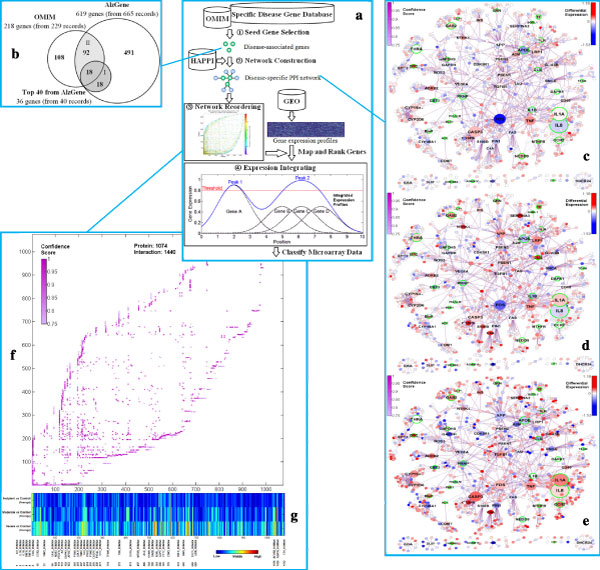
**An illustration for microarray classification by using integrative expression profiling (IXP) based on network reordering**. a) A framework for ACOR-based IXP approach. b) Overlap between Alzheimer's disease (AD) genes from OMIM and AlzGene databases. c-e) AD-specific PPI network layout with average differential expressions for three AD status (incipient, moderate, and severe) vs. control in GSE1297. Node size is gene weight, node color is differential expressions, and 36 I-class seed genes are greenly circled. f) The reordered adjacent matrix of the AD-specific PPI network. g) The corresponding average ACOR-based IXP profiles for the three contrasts.

A framework for microarray classification by using integrative expression profiling (IXP) approach based on network reordering (here we use ant colony optimization reordering - ACOR algorithm) is shown in Figure 1a. The ACOR-based IXP approach contains four steps: First, AD-associated genes are selected from AlzGene http://www.alzgene.org/ and OMIM http://www.ncbi.nlm.nih.gov/omim as seed genes. Second, an AD-specific protein-protein interaction (PPI) network is constructed by using nearest neighbor expansion algorithm [8] in an integrated human PPI database - human annotated and predicted protein interaction (HAPPI) [9]. Third, ACOR algorithm is applied in reordering the adjacency matrix of the constructed AD-specific PPI network. Finally, the gene expression profile for each sample is mapped to the ordered gene list, and integrated by using Gaussian function as influence function for each gene. The key step is to integrate gene expressions onto the gene list reordered from a disease-specific PPI networks by ACOR algorithm. As illustrated in the fourth step in Figure 1a, three closely ordered genes (B, C and D) form a new peak which is even greater than the peak formed by single gene (A) in integrated expression profiles. These three genes might be neglected by original expression profiling methods, due to their lowly differentially-expressed values. In our approach, if genes/proteins interact with each other, they will be put into neighboring orders. We use AD as an example to introduce the detailed methods and data sources in Additional file 1.

## Results and discussion

### AD-specific PPI network

We construct the AD-specific PPI network and visualize the network layout in Figure 1c-e. We also calculate the average differential expression values for the three AD status groups (incipient, moderate, and severe) vs. control group in GSE1297, and map them onto the genes in the network by representing them as node colors. There are 969 genes (90.2%) have expressions. From the comparisons of Figure 1c-e, we can see that differential expression increases from incipient to moderate, and then to severe AD status. This finding shows the validity of our network construction method, since this network is built specific for AD and the node color change directly reflects average gene expression shifts from incipient to severe AD. Moreover, not only hub genes (large sizes) and seed genes (green circled) are differentially expressed in different AD status, but also many non-hub genes (small sizes) surrounding hub genes are highly differentially expressed. This is the reason we could use IXP to make these "trivial" genes contribute the microarray classification.

### Reordered adjacent matrix

We use the ACOR algorithm under populated mode[4] to reorder the AD-specific PPI network. The reordered adjacency matrix is plotted in Figure 1f, which shows a fractal-like pattern also reported in another study on AD-specific PPI network, while using different seed genes [3]. The data indicate that the ACOR algorithm is robust on different seed gene selection and network construction processes. Since both the × and Y axes in Figure 1f denote reordering indexes (1-1074) of proteins, we also investigate the relative position for each protein. From the genes labeled in Figure 1g (with the same order of Figure 1f), we find almost all the I-class seed genes appear in the fringe of the left-bottom "head", while most II-class seed genes appear in the fringe of the "main body". This finding implies that the ACOR algorithm can not only group functionally related genes together (clustering capability), but also put them in a meaningful order (ranking capability). This combined characteristic (generating relative ranks in clusters, finally causing fractal-like patterns) is exactly what IXP needs. We also show that this order performs better than both classical ranking and clustering in microarray classification by IXP.

### Integrated expression profiles

We map the average differential expression values for the three AD status groups onto the gene list reordered by the ACOR algorithm. Then we integrate all the expression values for each group by using the IXP described by Equation (2) in Additional file 1. The integrated average expression profiles for the three AD status groups in GSE1297 are shown in Figure 1e. The profiles clearly indicate the distinctions among these three AD status groups and indicate the genes' differential expression increases from incipient to moderate, and then to severe AD status. This result not only verifies the usefulness of our MIXP method, but also validates our network construction method in a neater way than in network visualization.

### Classification performance comparisons

**Figure 2:**
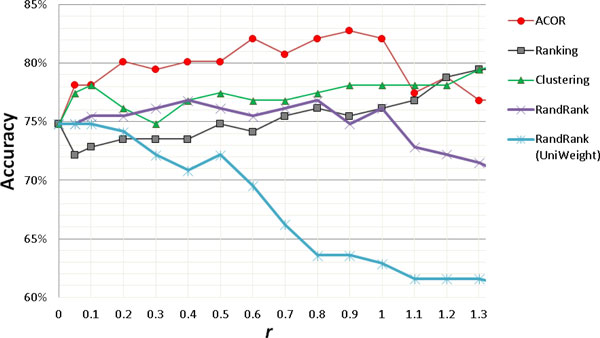
**Microarray classification performance comparisons for different integrative expression profiling (IXP) approaches**. Using GSE1297 as training set (31 samples, 22 AD patients vs. 9 controls), and GSE5281 (151 samples, 84 AD patients vs. 67 controls) as testing set, two-class (AD vs. control) classifications are performed for IXP approaches, based on ant colony optimization reordering (ACOR) algorithm, network ranking, graph clustering, and random-ordering (RandRank), with different coefficient ***r ***in Equation (2). UniWeight: unified gene weights (all equal to one).

By using GSE1297 as training set (31 samples, 22 AD patients vs. 9 controls), and GSE5281 (151 samples, 84 AD patients vs. 67 controls) as testing set, we perform two-class (AD vs. control) classifications for ACOR-based IXP approach with different horizontal influence coefficient *r *in Equation (2) (see Additional file 1). We also perform classifications for the IXP approaches based on network ranking [5] (similar with PageRank algorithm used by Google, equal to random walk ranking[10]), graph clustering [6] (2D hierarchical clustering, bioinformatics toolbox in Matlab), and on random-ordering (a random permutation of all network nodes), with different coefficient *r*. Here we use exactly the same gene weights calculated from node degree in the network to generate IXP profiles. The only difference here is the order of proteins in the network. As a comparison, IXP profiles based on the same permutation, but with unified gene weights (all equal to one), are generated. In Figure 2, the result for the blinded classification on GSE5281 shows that the ACOR-based IXP approach can improve accuracy from 74.83% (equal to *r *= 0) to 82.78% (*r *= 0.9) by using SVM classifier. A recently-published 1372-probe signature for AD [7] can only achieve 61.59% accuracy in the same condition (same training and testing sets, and same SVM classifier).

## Conclusions

From the blinded classifications on the testing microarray dataset with sample size 4 times bigger than the training microarray dataset from different microarray platforms, the ACOR-based IXP approach shows that it can serve as a knowledge-supervised feature transformation approach to increase classification accuracy dramatically, by transforming gene expression profiles to integrated expression files as features inputting into standard classifiers. The ACOR-based IXP approach also has better performance than the IXP approach based on ranking, clustering, and random-ordering. Since gene weights represent local topological properties and gene orders represent global topological characteristics, we find that both local and global network topology information can help IXP approach to improve classification accuracy. The order generated by ACOR algorithm provides the most help for sample classifications, a finding that implies the ACOR algorithm can group functionally related genes together in an ordered way.

## Competing interests

JYC and XW are co-founders of Medeolinx, LLC. This academic and private sector collaboration leverages Medeolinx's intellectual property, technologies, and equipment. However, this work does not promote any Medeolinx-related products or services.

## Authors' contributions

XW conducted network modeling and analysis. HH contributed to microarray analysis. MS participated in microarray classification. SR participated in gene cluster interpretation. JS participated in classification performance comparisons. JYC initiated the project and performed direct algorithm development, as well as contributed to the writing of the manuscript.

## Supplementary Material

Additional file 1 **Methods in detail**. Additional file describes the detailed methods and data sources used in this work. Click here for file
