# Supplementary material for: Reordering based integrative expression profiling for microarray classification
Source: BMC Bioinformatics. 2012 Mar 13;13(Suppl 2):S1. doi: 10.1186/1471-2105-13-S2-S1 (PMC3583189; doi:10.1186/1471-2105-13-S2-S1)
Supplement: Additional file 1 — Methods in detail. Additional file describes the detailed methods and data sources used in this work. [file 1471-2105-13-S2-S1-S1.docx]

# Methods in Details

## Microarray Datasets

The microarray datasets - GSE1297 (training set) and GSE5281 (testing set) in our case study on AD are downloaded both from GEO (http://www.ncbi.nlm.nih.gov/geo/). GSE1297 contains 20273 Probe IDs (mapped to 12679 genes) and 31 samples, divided into four groups - 9 healthy controls, 7 incipient AD patients, 8 moderate AD patients, and 7 severe AD patients. GSE5281 contains 40801 Probe Ids (mapped to 19700 genes) and 161 samples, from which, 151 samples (67 controls and 84 AD patients) are selected out after quality control. There are 12679 genes (gene symbol) overlapped between GSE1297 and GSE5281, which mapped to 12076 proteins (Uniprot ID), as features for classification. We use maximal expression values for same proteins mapped from different Probe IDs. We use Affy package in BioConductor for quantile normalization. For background correction, we use the built-in MicroArray Suite (MAS5).

## Seed Gene Selection

As shown in the first step in Figure 1a, disease-associated genes are selected from OMIM (http://www.ncbi.nlm.nih.gov/omim) and/or other specific disease gene databases, e.g. AlzGene (http://www.alzgene.org/) for AD-associated genes (simply, AD genes). In this study, we first choose 36 AD genes mapped from the Top 40 ranked genes/miRNAs/loci in AlzGene to Uniprot ID, as I-class seed genes. Then we choose 110 AD genes (Figure 1b) overlapped with 619 genes (mapped from 665 records till May 12, 2010) from AlzGene and 218 genes from OMIM (mapped from 229 records till August 19, 2010), as II-class seed genes. This approach assumes that these genes should show high significance both in literatures and GWAS studies on AD.

## Network Construction

To optimize computation time and information generation, we use a combined network construction strategy, based on both I-class and II-class AD seed genes. We first expand the 36 I-class seed genes in HAPPI [[1](#_ENREF_1)] with confidence score (*CI* >=0.75, i.e. 4-star rating) for interactions, by using nearest neighbor expansion (NNE) algorithm [[2](#_ENREF_2)], to obtain a PPI network with 516 proteins and 619 interactions. Then we expand the 110 II-class seed genes in HAPPI with confidence score (*CI* >=0.90, i.e. 5-star rating) for interactions, by using NNE algorithm, to obtain a PPI network with 755 proteins and 960 interactions. Finally, we combine these two networks to construct a node-weighted edge-scored AD-specific PPI network, containing 1074 proteins with node weights calculated by *W_i_* = {log_10_ (*Node_Degree_i_*)+1}/3, and 1440 interactions with edge scores for their confidence scores.

## Network Reordering

Ant colony optimization (ACO) [[3](#_ENREF_3)], is a dynamic stochastic searching (i.e. random walking [[4](#_ENREF_4)]) algorithm for finding optimal paths. The algorithm is based on the behavior of ants searching for food. ACO is also like another kind of dynamic method called “flow simulation”, which can be used as a graph clustering algorithm for the detection of protein families [[5](#_ENREF_5)], or as a classifying algorithm for the prediction of protein functions [[6](#_ENREF_6), [7](#_ENREF_7)].

To fulfill the task of network reordering, we use the ant colony optimization reordering (ACOR) algorithm under populated mode [[8](#_ENREF_8), [9](#_ENREF_9)], in which, simulated ants (*s-ant*) roam all possible network paths iteratively and populate quickly (total population of s-ant colony increases rapidly). As shown in Equation (1), the iteration process can be manipulated to get the density distribution *s_i_* of s-ants crowding on each node. According to this density distribution *s_i_*, the reordered adjacency matrix of the network is shown as a heat map to reveal the system-level features of the network.

$\begin{matrix} s_{i+1}=\mathbf{P}_{i}\times s_{i},\mathbf{P}_{i}\in R^{n\times n},s_{i}\in R^{n},s_{0}=(1/n,1/n,...,1/n)^{T} \\ \mathbf{P}_{i+1}=\mathbf{P}_{i}(c_{i},c_{i}),c_{i}=\text{Ordering}(s_{i}),i=0,1,...,N-1 \end{matrix}$ (1)

Here *s_i_* denotes *i* th step density distribution of s-ants crowding on each node, P is the adjacency matrix of a network, *c_i_* is a permutation vector according to *i* th step density distribution *s_i_*, and P*_i_* (*c_i_*, *c_i_*) is the reordered adjacency matrix with the permutation *c_i_*. The iteration process will be manipulated until the permutation *c_i_* changes little. Both the network reordering and followed expression integration processes are programed by using Matlab.

## Expression Integrating

The gene expression profile for each sample is mapped onto the gene list reordered by the ACOR algorithm under populated mode. The integrated expression profile *IXP*(*t*) is calculated by simply using Gaussian function as an influence function for each gene, and then by adding up the influence functions from all the genes together, as shown in Equation (2).

$IXP(t)=\sum_{i=1}^{L} |E_{i}|e^{-\frac{1}{rW_{i}}(t-i)^{2}},t=1,...,L$ (2)

Here *L* is the length of the gene list, and *r* is a horizontal influence coefficient for all genes. The normalized gene expression value *E_i_* determines the vertical influence of gene *i*. The weight value *W_i_* is calculated from node degree as described in Network Construction Section, which determines the horizontal influence of gene *i*. An illustration for this function can also be found in the fourth Step in Figure 1a.

## Microarray Classification

Support vector machine (SVM) type 2 with linear kernel is used through all the microarray classifications here. Before classification, all the inputted features (gene expression values for each sample) are scaled to normal distribution with zero mean and one standard deviation. Matlab bioinformatics toolbox is used for programming.

# References

1. Chen JY, Mamidipalli S, Huan T: **HAPPI: an online database of comprehensive human annotated and predicted protein interactions**. *BMC Genomics* 2009, **10 Suppl 1**:S16.

2. Chen JY, Shen C, Sivachenko AY: **Mining Alzheimer disease relevant proteins from integrated protein interactome data**. *Pac Symp Biocomput* 2006:367-378.

3. Dorigo M, Bonabeau E, Theraulaz G: **Ant algorithms and stigmergy**. *FUTURE GENER COMPUT SYST* 2000, **16**(8):851-871.

4. Kohler S, Bauer S, Horn D, Robinson PN: **Walking the interactome for prioritization of candidate disease genes**. *The American Journal of Human Genetics* 2008, **82**(4):949-958.

5. Enright AJ, Van Dongen S, Ouzounis CA: **An efficient algorithm for large-scale detection of protein families**. *Nucleic Acids Research* 2002, **30**(7):1575-1584.

6. Nabieva E, Jim K, Agarwal A, Chazelle B, Singh M: **Whole-proteome prediction of protein function via graph-theoretic analysis of interaction maps**. *Bioinformatics* 2005, **21**(1):i302-i310.

7. Chua HN, Sung WK, Wong L: **Exploiting indirect neighbours and topological weight to predict protein function from protein-protein interactions**. *Bioinformatics* 2006, **22**(13):1623-1630.

8. Wu X, Huan T, Pandey R, Zhou T, Chen JY: **Finding fractal patterns in molecular interaction networks: a case study in Alzheimer's disease**. *International Journal of Computational Biology and Drug Design* 2009, **2**(4):340-352.

9. Wu X, Pandey R, Chen JY: **Network topological reordering revealing systemic patterns in yeast protein interaction networks**. *IEEE Engineering in Medicine and Biology Society* 2009, **1**:6954-6957
